# A Longitudinal Network Analysis of Depressive Symptoms Among Older Adults: Findings From an 8‐Year Prospective China National Survey

**DOI:** 10.1155/da/3846758

**Published:** 2026-01-07

**Authors:** Meng-Yi Chen, He-Li Sun, Yuan Feng, Qinge Zhang, Zhaohui Su, Teris Cheung, Matteo Malgaroli, Todd Jackson, Yu-Tao Xiang

**Affiliations:** ^1^ Unit of Psychiatry, Department of Public Health and Medicinal Administration, Institute of Translational Medicine, Faculty of Health Sciences, University of Macau, Macao SAR, China, umac.mo; ^2^ Centre for Cognitive and Brain Sciences, University of Macau, Macao SAR, China, umac.mo; ^3^ College of Acupuncture-Moxibustion and Tuina, International Institute for Innovation in Acupuncture, Beijing University of Chinese Medicine, Beijing, China, bucm.edu.cn; ^4^ Beijing Key Laboratory of Mental Disorders, National Clinical Research Center for Mental Disorders and National Center for Mental Disorders, Beijing Anding Hospital, Capital Medical University, Beijing, China, ccmu.edu.cn; ^5^ School of Public Health, Southeast University, Nanjing, China, seu.edu.cn; ^6^ School of Nursing, Hong Kong Polytechnic University, Hong Kong SAR, China, polyu.edu.hk; ^7^ Department of Psychiatry, NYU Grossman School of Medicine, New York, USA, med.nyu.edu; ^8^ Department of Psychology, University of Macau, Macao SAR, China, umac.mo

**Keywords:** depression, longitudinal study, network analysis, older adults

## Abstract

**Background:**

Late‐life depression (LLD) is a significant global public health challenge among older adults. Exploring central/influential symptoms with longitudinal study designs can enhance the efficacy of detection, early prevention, and interventions for LLD. This study aimed to identify key symptoms of LLD using a panel graphical vector autoregression (panel‐GVAR) model based on longitudinal national survey data.

**Methods:**

Data from the China Health and Retirement Longitudinal Study (CHARLS) between 2013 and 2020, encompassing four waves, were utilized to construct a longitudinal depressive symptom network. Depressive symptoms were assessed using the 10‐item Center for Epidemiological Studies Depression Scale (CESD‐10). In expected influence (in‐EI) and out expected influence (out‐EI) were identified to characterize the interaction of symptoms within the temporal network, while expected influence (EI) was used to examine the interaction of symptoms in both the contemporaneous network and the between‐subjects network.

**Results:**

A total of 1393 older adults were assessed. A persistently significant increase in the prevalence of depression was observed over time. In the temporal network, “restless sleep” (CESD7) and “could not get going” (CESD10) were the most influential symptom and most influenced symptom, respectively. In both the contemporaneous network and the between‐subjects network, “felt depressed” (CESD3) emerged as the most central symptom within the community of depressive symptoms.

**Conclusions:**

Given the challenges associated with treating LLD and its adverse effects on daily life for older adults, timely interventions targeting identified key symptoms may help prevent and mitigate depression in this population.

## 1. Introduction

Depression represents a widespread psychological condition that substantially impacts global health burdens through increased disability and death rates [1]. When occurring in older adults, this condition is termed late‐life depression (LLD), characterized by depressive episodes in individuals of advanced age [2]. With the ongoing demographic shift toward older populations internationally, LLD has emerged as a critical issue in both healthcare systems and community health initiatives [3]. Research reveals that LLD affects between 8.2% and 63.1% of older adults globally, with a weighted average prevalence of 28.4% (95%CI: 24.8%−32.0% [4]). China, home to the world’s largest older adults’ group, reports LLD rates of 30.6% among its senior citizens [5], while diagnostic assessments using the Fourth Edition of the Diagnostic and Statistical Manual of Mental Disorders (DSM‐IV) criteria identify major depressive disorder (MDD) in 20.3% of this population [6]. Scientific investigations have established connections between LLD and numerous negative health consequences like obesity, frailty, and cognitive decline [7, 8]. LLD also frequently coexists with physical disorders, including heart disease, stroke, metabolic disorders, and neurodegenerative diseases [8–11]. Left untreated, LLD can severely diminish older adults’ physical wellbeing, daily functioning, and overall life satisfaction [12].

In recent years, network analysis, an advanced method of data analysis, has gained growing attention in the fields of psychiatry and psychology [13]. A significant advantage of network analysis is its capacity to reveal the complexity of psychological structures and demonstrate how various symptoms interact with each other [14]. Network approaches assume that mental health problems often arise from a web of interrelated symptoms rather than isolated ones and allow for the examination of psychiatric disorders or syndromes at the level of individual symptoms, including interactions among multiple symptoms [15]. Network analysis visually represents specific symptoms as nodes and interactions between symptoms as edges through organized spatial networks [16]. The positioning of nodes indicates the significance of individual symptoms, while the thickness of edges reflects the strength of connections between symptoms [17]. By identifying and prioritizing the most central symptoms (i.e., hypothesized core drivers) of psychiatric disorders or syndromes, interventions can be tailored to reduce risk of relapse and symptom severity [18]. Network analysis has been used extensively in studying LLD; for instance, research on older Korean adults revealed that “felt unhappy”, “hopelessness,” and “emptiness” were the most influential symptoms in this population [19]. Similarly, in a study of Australian older adults with cancer, “anhedonia,” “sad mood,” and “guilt” emerged as central symptoms in the associated symptom network [20]. More broadly, a review on MDD concluded that “depressive mood” and “fatigue” play a critical role in network models of older adults [21]. According to socioemotional selectivity theory [22], older adults perceive their future time to be limited and, therefore, often prioritize emotionally rewarding and meaningful social relationships; depressive symptoms reflect the frustration of this goal. Among key depressive symptoms, fatigue often restricts participation in activities of interest, while mood‐related symptoms directly conflict with the emotion‐focused objectives of older adults, ultimately contributing to LLD.

However, traditional network analyses of depression have several common limitations. In particular, most studies have been based on cross‐sectional data, making it difficult to determine the temporal continuity and conductive orientation of symptoms. This drawback limits clinical applications, as most appropriate early interventions for central symptoms may not be easily derived from cross‐sectional data [23]. Cross‐lagged panel network (CLPN) longitudinal analysis was developed to address this problem, but model establishment based on this method is limited to two time points and cannot incorporate multiple time points. Consequently, the randomness of results is large [24]. Furthermore, clinical symptoms or nodes can interact differently over time at individual versus group levels. For example, the associations between “exercise” and “heart rate” at the individual (within‐subject) level are positively associated with each other, as heart rate typically increases during high‐intensity exercise. However, while at a between‐subjects group level, “exercise” and “heart rate” can be negatively associated with each other, as people who engage in more exercise generally exhibit slower average heart rates [25]. This example illustrates how central symptoms derived from traditional group‐based network analysis may not be applicable to specific individuals. In fact, neither traditional cross‐sectional network analysis nor CLPN longitudinal network analysis can solve the problem of cross‐dimensional interactions of symptoms. Towards addressing this limitation, multidimensional modeling and its application to practical clinical problems have become the focus for technological advancements in network analysis [26].

Panel graphical vector autoregression (panel‐GVAR) model has been introduced into network analysis recently to address these limitations. Panel‐GVAR was initially developed in econometrics and used to analyze relationships among multiple time series variables within a panel dataset [27]. This approach extends the traditional vector autoregression (VAR) model by incorporating cross‐sectional dependencies among different participants in a panel setting [28]. The term “panel” refers to data from multiple individuals or groups, each tracked over time. Panel‐GVAR utilizes multiple time points, incorporating time‐lagged effects through the VAR structure. The approach also accounts for group‐level influences and individual dynamic changes [29]. In a panel‐GVAR model, each participant is represented by a vector of variables, and interactions among participants are captured through three distinct networks: a temporal network, a contemporaneous network, and a between‐subjects network [25]. These networks allow for a simultaneous investigation of longitudinal interactions, individual‐level interactions, and group‐level interactions of symptoms, providing a comprehensive perspective on the occurrence, progression, and translation of psychiatric syndromes [30].

To the best of our knowledge, no studies to date have employed the panel‐GVAR network perspective to explore the most central/influential symptoms of LLD. To address this gap and better clarify the most central symptoms of LLD, we conducted a longitudinal network analysis study on depressive symptoms among older adults based on the China Health and Retirement Longitudinal Study (CHARLS).

## 2. Methods

### 2.1. Data Source and Study Population

This research utilized existing data from the CHARLS, a nationwide prospective investigation targeting individuals aged 45 and above living in community settings [31]. Launched in 2011, CHARLS conducted subsequent waves of data collection in 2013, 2015, 2018, and 2020 [32]. To enhance sample representativeness, researchers employed a multistage sampling approach, selecting participants from 450 rural villages and urban neighborhoods within 150 county‐level administrative units distributed across 28 Chinese provinces [31]. Comprehensive information regarding the study’s methodology, implementation, and sampling strategy has been published elsewhere [33]. Ethical clearance for the CHARLS project was obtained from Peking University’s Biomedical Ethics Review Board (approval number IRB00001052‐11015), with all participants providing signed consent forms prior to their involvement.

Regarding the CHARLS, trained undergraduate and graduate students were interviewers, whose competence was ensured through rigorous instruction, assessments and mock interviews. Data were collected face‐to‐face using computer‐assisted personal interviewing (CAPI) that allowed real‐time error detection and correction. If participants were unavailable due to health or cognitive limitations, a proxy who was familiar with participants responded on their behalf. Field supervisors monitored data collection to address operational challenges. Further methodological details, including quality control protocols, are available in previous publications [34, 35].

**Figure 1:**
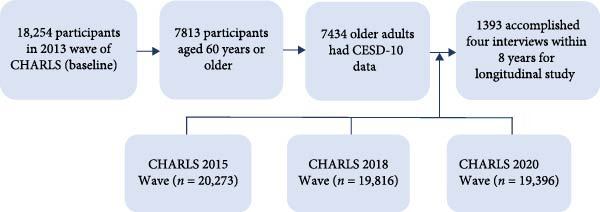
The flowchart of this study. CESD‐10, 10‐item Center for Epidemiological Studies Depression Scale; CHARLS, China health and retirement longitudinal study.

This investigation employed longitudinal data from four CHARLS survey waves (2013‐Wave 2, 2015‐Wave 3, 2018‐Wave 4, and 2020‐Wave 5), with the initial 2013 measurements serving as the “baseline assessment.” The analytical approach involved developing a dynamic symptom network through panel‐GVAR [26, 36]. Eligible participants met three key criteria: (1) continuous participation across all four survey waves, (2) completion of required depression evaluations at each timepoint, and (3) age of at least 60 years during the baseline 2013 assessment. Figure 1 illustrates the complete participant selection process.

All eligible older adults were included in the longitudinal network analysis. A Cochran–Armitage trend test was used to assess changes in the proportion of older adults with depression over the study period between 2013 and 2020 [37].

### 2.2. Measurement Tools

Severity of depressive symptoms was measured using the 10‐item Chinese version of the Center for Epidemiological Studies Depression Scale (CESD‐10) [38, 39]. CESD‐10 items include: (1) bothered by things; (2) trouble concentrating; (3) felt depressed; (4) everything was an effort; (5) hopeless about future; (6) felt fearful; (7) restless sleep; (8) felt unhappy; (9) felt lonely; 10) could not get going. The CESD‐10 includes two reverse‐scored items (items 5 and 8). Original CESD‐10 items were scored from “0” (rarely or none of the time) to “3” (most or all of the time), while reverse‐scored items were scored from “0” (most or all of the time) to “3” (rarely or none of the time). Total scores ranged from 0 to 30, with higher total CESD‐10 scores reflecting more severe depressive symptoms; a CESD‐10 total score of ≥10 indicates “having depression” [40]. The Chinese version of the CESD‐10 has been widely used and validated with satisfactory psychometric properties (e.g., Cronbach’s alpha of *α* = 0.815) [41].

Sociodemographic characteristics of the sample we assessed included age, gender, marital status, education, residential location, religious belief, and comorbid physical diseases. The listwise deletion method was used to address item‐level and wave‐level missing data.

### 2.3. Longitudinal Network Analysis

Longitudinal network analyses were performed using R software (version 4.3.3) [42]. Panel‐GVAR was carried out to unravel interactions of depressive symptoms across the four waves of CHARLS using the psychonetrics package (version 0.13) [43]. First, a raw Panel‐GVAR model was estimated, followed by a pruned model and a step‐up iterative model, where individual edges were added and/or pruned using a threshold of *α* = 0.05. Bayesian Information Criteria (BIC) were applied to identify the best fitting panel‐GVAR [25]. R package qgraph (version 1.9.8) was used for network visualization [44].

The panel‐GVAR model included three networks, a temporal network, a contemporaneous network, and a between‐subjects network. In these network models, individual symptoms were visualized as nodes, while relationships between symptoms were visualized as edges. A temporal network can depict how one single node (i.e., symptom) at a later time point was predicted by other nodes at a preceding time point via a regression coefficient directed network, after controlling for individual symptom differences of all participants from the initial wave [30]. Next, after controlling for time differences and any other variables within the same assessment wave, remaining variances and covariances can be modeled as a graphical Gaussian model (GGM), i.e., contemporaneous network. Contemporaneous network reflects within‐subject contemporaneous effects of a personalized undirected network within the same external environment and time conditions [45]. Finally, a between‐subjects network is essentially a cross‐sectional network of participant‐level means of all variables and can reveal how individual symptoms are associated with one another across participants, without taking time into consideration [30].

Regarding centrality indexes, the contemporaneous network and the between‐subjects network relied on one indicator: expected influence (EI). For each node, EI represents the summed weight of all associated edges with this node (i.e., symptom) in a partial correlated network, including positive and negative associations [25]. Blue and red color edges reflect positive and negative correlations, respectively. In contrast, the temporal network had two indicators: in EI (in‐EI) and out EI (out‐EI). For each node, In‐EI indicates the extent to which a symptom is predicted by other symptoms (i.e., the sum of values of incoming edges associated with one symptom), while out‐EI denotes the extent to which a symptom can predict other symptoms (i.e., the sum of values of outgoing edges associated with one symptom) [46]. Directed edges of each node pointing to itself signify autoregressive coefficients, while directed edges pointing to other nodes signify panel‐GVAR prediction effects. Arrow colors represented directions of effects, with blue arrows indicating positive effects and red arrows indicating negative effects. By establishing a variable/node regression model via the temporal network, variable autoregression and interact effects were assessed by all variables at a latter time observation point (*t*) onto a previous time observation point (*t*‐1) [30, 47]. In all three network models, higher EI values indicated stronger prediction effects/more importance, while thicker edges reflected stronger associations.

## 3. Results

### 3.1. Study Sample

The baseline analysis incorporated data of 1393 older adults from the 2013 CHARLS cohort. As presented in Table 1, the sample had an average age of 67.77 years (standard deviation [SD = 5.64], with 54.63% [*n* = 761] being male, 80.11% [*n* = 1,116] married, and 22.97% [*n* = 320]) having attained at least a secondary education. The mean CESD‐10 total score was 8.47 (SD = 5.73), and 36.61% (*n* = 510, 95% CI: 34.08%–39.14%) met the cutoff for depression (CESD‐10 ≥ 10). A comparison between included and excluded older adults (Table S1) revealed no statistically significant differences in demographic or clinical variables.

**Table 1:** Demographic and clinical characteristics of participants at the baseline (*N* = 1393).

Variables	*N*	%
Male	761	54.63
Married	1116	80.11
Education		
Primary school and below	1073	77.03
Secondary school and above	320	22.97
Urban residence^a^	196	14.07
Having religious belief	170	12.20
Having two or more major physical diseases^a^	221	15.87
CESD‐10 total ≥ 10	510	36.61

**Variables**	**Mean**	**SD**

Age (years)	67.77	5.64
CESD‐10 total	8.47	5.73

*Note:* CESD‐10, 10‐item center for epidemiological studies depression scale.

Abbreviaton: SD, standard deviation.

^a^The frequencies are calculated based on available data.

### 3.2. Change Trend of Prevalence of Depression

**Figure 2:**
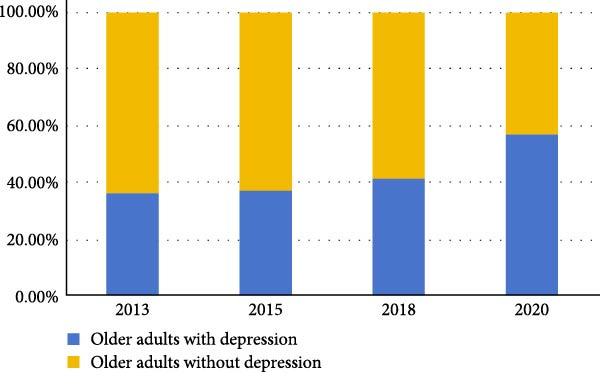
Longitudinal change trend of the proportion of older adults with depression.

Figure 2 shows the 8‐year change trend of the proportion of older adults with depression. Prevalence rates of depression in 2013, 2015, 2018, 2020 were 36.61% (95%CI:34.08%−39.14%), 37.26% (95%CI:34.72%−39.80%), 41.71% (95%CI:39.12%−44.30%), and 57.29% (95%CI:54.69%−59.89%), respectively. The Cochran–Armitage trend test demonstrated a statistically significant increase in the prevalence of depression over time (*Z* = 6.910, *p*‐value < 0.001).

### 3.3. Longitudinal Network Analysis of Older Adults

**Figure 3:**
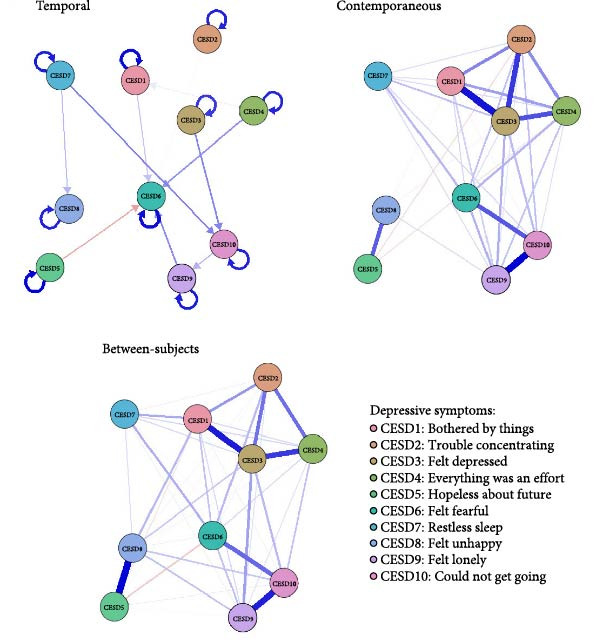
Temporal, contemporaneous, and between‐subjects networks of depressive symptoms. CESD‐10, 10‐item Center for Epidemiological Studies Depression scale.

**Figure 4:**
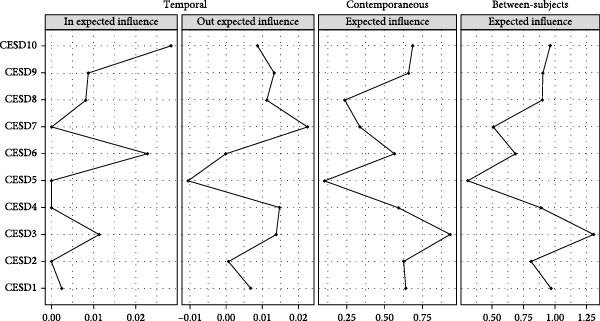
The centrality of depressive symptoms in the network of temporal, contemporaneous, and between‐subjects. CESD‐10, 10‐item Center for Epidemiological Studies Depression Scale. CESD1, bothered by things; CESD2, trouble concentrating; CESD3, felt depressed; CESD4, everything was an effort; CESD5, hopeless about future; CESD6, felt fearful; CESD7, restless sleep; CESD8, felt unhappy; CESD9, felt lonely; CESD10, could not get going.

Model fit information for the Panel‐GVAR network model is reported in Table S2. Figure 3 shows the panel‐GVAR models of depressive symptoms, which included temporal, contemporaneous, and between‐subjects networks. In the temporal network depicting interactions of symptoms over time, the strongest association was CESD7 ‐> CESD10 (“restless sleep” to “could not get going”, edge weight = 0.0144). In terms of autoregressions, all 10 depressive symptoms demonstrated positive autoregressive pathways, with CESD5 (“hopeless about future,” edge weight = 0.2879) emerging as the most prominent predictor; higher past “hopeless about future” severity strongly predicted future occurrences of the same symptom among older adults. For centrality indices, CESD7 (“restless sleep”, out‐EI = 0.0225) exhibited the highest out‐EI value, reflecting a comparatively stronger effect in triggering other depressive symptoms in the future. Conversely, CESD10 (“could not get going”, in‐EI = 0.0283) was identified as the symptom with the highest in‐EI value, indicating that the onset and/or worsening of other depressive symptoms were most likely to trigger the onset and worsening of CESD10 (“could not get going”) at a subsequent time (Figure 4).

In the contemporaneous network, the strongest association was CESD9–CESD10 (“felt lonely” to “could not get going”, edge weight = 0.2746). For the centrality indices, CESD3 (“felt depressed”, EI = 0.9349) had the highest EI value, indicating that, for individual older adults, “felt depressed” was the most central symptom (indicator) for the onset and maintenance of other depressive symptoms in the model.

Regarding the between‐subjects network, the strongest association was for CESD5–CESD8 (“hopeless about future” to “felt unhappy”, edge weight = 0.4017). For the centrality indices, CESD3 (“felt depressed”, EI = 1.3081) exhibited the highest EI value, indicating that for this sample of older adults, “felt depressed” was a more important symptom for holistic prevention and intervention of depressive symptoms.

## 4. Discussion

To our knowledge, this is the first study to employ panel‐GVAR models using longitudinal network analysis to examine depression in older adults. The increased prevalence of depression among Chinese older adults is a concerning trend observed over an 8‐year period. In the temporal network, “restless sleep” emerged as the most influential symptom related to the risk of potential future depressive symptoms. Conversely, “could not get going” was identified as the most influenced symptom, often following the onset of other depressive symptoms. In both the contemporaneous network and between‐subjects network, “felt depressed” was the most central symptom, highlighting its significant role in the symptom community of LLD at both individual and group levels. Implications of these findings are discussed below.

The increased prevalence of depression over time in older adults is in line with research on LLD in low‐ and middle‐income countries [48]. According to existing trajectory research on LLD, depressive symptoms of most older adults show a continuous worsening trend, particularly after the age of 70, which partially aligns with findings of this study [49]. Some authors have contended that depression and aging are mutually reinforcing [50, 51]. On one hand, depression could exacerbate aging due to its associations with increased oxidative stress and neuroendocrine system dysfunction [52, 53]. On the other hand, some changes brought about by aging, such as cognitive decline, chronic physical diseases, interpersonal losses, social isolation, and disturbances in self‐identity and self‐worth, are risk factors for LLD [54, 55].

In addition, other factors could exacerbate LLD. For example, the rapid urbanization process in China in recent years has accelerated the centralization of family structures, potentially increasing risk for depression among empty‐nest elders [56]. Furthermore, primary healthcare resources in China, especially those related to mental health treatment, are limited. Consequently, interventions to prevent exacerbations in depressive symptoms of older adults are not easily accessible for many older Chinese adults [57]. Therefore, early interventions for depression among older adults are of great significance. Treatments targeting physical health, cognitive functioning, social support, well‐being, and overall satisfaction with life in older adults [58] may have utility in reducing depression risk and incidence.

“Restless sleep” emerged as the most influential symptom of LLD in the temporal network. Based on arrow directions, “restless sleep” was related mainly to “felt unhappy” and “could not get going” in subsequent waves. Previous findings from traditional network analysis showed that “restless sleep” served as a key central symptom of depression across various age groups, including adolescents, women and older adults [59–61]. Several factors might contribute to this finding. First, prolonged restless sleep might disrupt the release and activity of neurotransmitters related to emotion regulation, such as serotonin and dopamine [62, 63], which are crucial for maintaining a healthy emotional state [64]. Second, persistent restless sleep could exacerbate emotional burdens and cognitive decline, leading to feelings of increased fatigue and irritability [65], which may impair decision‐making and emotion regulation [66]. Finally, restless sleep could disrupt circadian rhythms and cause physical and mental discomfort, ultimately increasing risk for depression [67, 68]. Therefore, for older adults experiencing restless sleep, timely interventions such as internet‐based cognitive–behavioral therapy (ICBT) and behavioral sleep hygiene strategies warrant consideration in helping to prevent the onset and progression of other depressive symptoms [69].

“Could not get going” was identified as the most influenced depressive symptom within the temporal network. Based on arrow directions, “could not get going” was related principally to previous “felt depressed” and “restless sleep” symptoms from earlier waves. “Could not get going” has been recognized as a bridge symptom linking depression and other mental health problems among older adults, including loneliness, anxiety, and insomnia [70–72]. “Could not get going” refers to a lack of motivation and energy, sometimes described as “lack of energy” in the literature [73]. Compared to younger individuals, older adults tend to exhibit more atypical depressive symptoms [74] and suffer from changes brought about by aging such as the loss of social ties, reduced activities, a slower pace of life, and chronic illnesses [75]. Some researchers regard “could not get going“/”lack of energy” as a normative physiological change that comes with aging rather than a depressive symptom for older adults [76, 77]. However, recent studies have found that energy plays an important role in the mental health of the older adults, contributing to enhanced self‐confidence, independence and autonomy, reduced feelings of social isolation, better quality of life, and success in fending off stressors and side effects of antidepressant treatment [78]. For older adults with chronic physical diseases and comorbid depression, sufficient energy contributes to improved overall physical and mental well‐being [79]. Furthermore, temporal network results suggest that improvements in energy and motivation may be a useful marker for evaluating the effectiveness of LLD interventions and treatment, particularly when controlling for the influence of physical diseases and normative changes in aging, as other depressive symptoms are most likely to trigger subsequent “could not get going” symptoms.

“Felt depressed” emerged as the most central symptom in both contemporaneous network and between‐participants network models, underscoring its crucial role in triggering other depressive symptoms at both individual and group levels [80]. This finding aligns with several previous cross‐sectional network analyses. For instance, a study of older adults in the United States identified “felt depressed” as the most central symptom of depression both before and after the onset of chronic diseases [81]. Similarly, research involving Chinese older adults found “felt depressed” to be the most central symptom of depression [82]. According to the diagnostic guidelines for psychiatric disorders from the American Psychological Association (APA) and World Health Organization (WHO), persistent “felt depressed” is a key indicator and characteristic symptom of MDD [83, 84]. The impact of “felt depressed” is profound across both physical and mental domains of older adults and can affect cognitive function, promote negative emotions and exacerbate other depressive symptoms [85, 86]. In addition, depressed mood may hinder emotional expression and social interactions, contributing to social detachment and loneliness, ultimately worsening overall depressive symptomatology [87]. Appetite, sleep quality, and physical vigor can also be affected by depressed mood, compromising quality of life and increasing the risk of other depressive symptoms [88–90]. Therefore, timely interventions targeting “felt depressed” in older adults may be imperative to preventing the escalation of other depressive symptoms.

In sum, key symptoms identified in the panel‐GVAR model have potential implications for clinical practice. For example, the most influential temporal network symptom, “restless sleep”, underscores potentially relevant interventions (e.g., sleep hygiene) that might help to control and manage the occurrence and development of other depressive symptoms, particularly in early stages of depression. The most influenced temporal network symptom, “could not get going”, could serve as a possible indicator of depression intervention effectiveness; ongoing loss of energy during or following treatment might indicate other depressive symptoms have not been adequately addressed. The identification of “felt depressed” as the most central symptom across contemporaneous and between‐subjects networks, suggested its crucial role in the entire depressive symptom community of older adults. Relevant targeted interventions for these symptoms should be tested in research on prevention and treatment of LLD, as well as daily management. Furthermore, the autoregression effect from the panel‐GVAR model also had specific clinical implications. Within this model, “hopeless about future” had the highest temporal network autoregression value, suggesting that once older adults develop hopelessness about later life, such feelings might persist or reemerge in the future. Therefore, for older adults with a history of depressive episodes, monitoring, and intervening to address hopelessness is important for improving well‐being.

Although findings provide empirically‐grounded hypotheses for future treatment research, it is also important to acknowledge that this prioritization of symptoms is derived from a core tenet of network analysis, the contention that symptoms with the highest centrality warrant primary intervention [73]. While there is a theoretical rationale for this assumption, the practical efficacy of such an approach must ultimately be validated through rigorous clinical research.

Strengths of this study included its nationally representative sample, analyses of longitudinal data assessed over multiple timepoints during the course of 8 years, and the employment of a novel panel‐GVAR model to provide a distinct perspective on the LLD symptom network. However, several limitations should be acknowledged. First, the sample size of this study was relatively small, in part, because the establishment of a panel‐GVAR model requires data spanning multiple observation points, thereby increasing the likelihood of attrition of many potential participants and limiting statistical power. Second, listwise deletion was employed to address missing CHARLS data. This method can reduce statistical power by excluding partial observations and introduce selection bias if missing data are not completely random, potentially affecting the generalizability of findings. That said, it was also somewhat reassuring that included versus excluded participant subsets did not differ statistically on any demographic or clinical background measures. Third, the generalizability of findings may be limited to Chinese community‐dwelling older adults and may not extend to older adults in other countries or older cohorts who are hospitalized for physical and mental health problems. Fourth, because adjacent waves had a minimum time interval of 2 years, certain short‐term associations between symptoms may have been masked given that symptom‐level fluctuations within 2 years could not be captured. Fifth, consistent with limitations of other published research using panel‐GVAR [30, 47], there is currently no consensus regarding objective sensitivity analysis for this approach. Therefore, the stability and accuracy of this network model require further validation through future technological advancements and clinical studies. Finally, while promising, evidence for such network‐derived interventions is preliminary. Although our results provided empirically‐based hypotheses for specific intervention targets, clinical benefits of such interventions should be rigorously tested in future intervention research.

## 5. Conclusion

In conclusion, the prevalence of depression persistently increased over 8 years among older Chinese adults. “Restless sleep” was identified as the most influential symptom for the emergence and aggravation of other depressive symptoms in the future, while “could not get going” was the most influenced symptom and could serve as a marker for intervention efficacy in reducing depression symptoms. “Felt depressed” was the most central symptom of depression both at individual and group levels; timely intervention targeting this symptom may be especially conducive to the prevention and management of other depressive symptoms among older adults.

## Conflicts of Interest

The authors declare no conflicts of interest.

## Author Contributions

Study design: Meng‐Yi Chen, Yuan Feng, Qinge Zhang, Yu‐Tao Xiang. Data collection, analysis, and interpretation: Meng‐Yi Chen, He‐Li Sun, Zhaohui Su, Teris Cheung, Matteo Malgaroli. Drafting of the manuscript: Meng‐Yi Chen, Yu‐Tao Xiang. Critical revision of the manuscript: Todd Jackson. Meng‐Yi Chen, He‐Li Sun, Yuan Feng, and Qinge Zhang contributed equally to this work.

## Funding

The study was supported by the Beijing High Level Public Health Technology Talent Construction Project (Discipline Backbone‐01‐028), the Beijing Municipal Science & Technology Commission (Grant Z181100001518005), the Beijing Hospitals Authority Clinical Medicine Development of Special Funding Support (XMLX202128), and the University of Macau (Grants MYRG2019‐00066‐FHS and MYRG2022‐00187‐FHS).

## Supporting Information

Additional supporting information can be found online in the Supporting Information section.

## Supporting information


**Supporting Information** Table S1: Comparison of characteristics of the excluded and included older adults was presented in Table S1. A comparison between included and excluded older adults revealed no statistically significant differences in demographic or clinical variables. Table S2: Model fit information for Panel‐GVAR network model of depression symptoms among older adults was presented in Table S2.

## Data Availability

All data supporting the findings of this study was available in the Peking University Open Platform: China Health and Retirement Longitudinal Study (CHARLS, http://charls.pku.edu.cn/en).
