# Supplementary material for: A Longitudinal Network Analysis of Depressive Symptoms Among Older Adults: Findings From an 8‐Year Prospective China National Survey
Source: Depress Anxiety. 2026 Jan 7;2026:3846758. doi: 10.1155/da/3846758 (PMC12777696; doi:10.1155/da/3846758)
Supplement: Supplementary file 1 — Supporting Information Table S1: Comparison of characteristics of the excluded and included older adults was presented in Table S1. A comparison between included and excluded older adults revealed no statistically significant differences in demographic or clinical variables. Table S2: Model fit information for Panel‐GVAR network model of depression symptoms among older adults was presented in Table S2. [file DA-2026-3846758-s001.docx]

**Supplementary material**

**Table S1.** Comparison of characteristics of the excluded (n=6,420) and included older adults (n=1,393)

**Table S2.** Model fit information for Panel-GVAR network model of depression symptoms among older adults (n = 1,393)

**Table S1.** Comparison of characteristics of the excluded (n=6,420) and included older adults (n=1,393)

| Variable | Included older adults (n=1,393) | | Excluded older adults (n=6,420) | | | Univariable analysis | | | |
| --- | --- | --- | --- | --- | --- | --- | --- | --- | --- |
|  | n | % | | n | % | | X^2^ | df | p |
| Male | 761 | 54.63 | | 3,343 | 52.07 | | 3.005 | 1 | 0.083 |
| Married | 1,116 | 80.1 | | 5,097 | 79.4 | | 0.443 | 1 | 0.505 |
| Education (Secondary school and above) | 1,073 | 77.03 | | 5,046 | 78.60 | | 1.816 | 1 | 0.178 |
| Urban residence* | 196 | 14.07 | | 864 | 13.50 | | 0.319 | 1 | 0.572 |
| Having religious belief | 170 | 12.20 | | 828 | 12.90 | | 0.494 | 1 | 0.482 |
| Having two or more major physical diseases* | 221 | 15.87 | | 1,098 | 17.12 | | 1.138 | 1 | 0.286 |
|  |  |  | |  |  | |  |  |  |
| Variable | Mean | SD | | Mean | SD | | Z | df | p |
| Age (years) | 67.77 | 5.64 | | 67.61 | 5.73 | | -0.938 | - | 0.348 |

Note: The excluded older adults group consists of 379 individuals with missed data and 6,041 individuals lost to follow-up.

* The frequencies are calculated based on available data.

**Table S2.** Model fit information for Panel-GVAR network model of depression symptoms among older adults (n = 1,393)

|  |  | BIC | AIC | RMSEA |
| --- | --- | --- | --- | --- |
| Baseline model |  | 150463.31 | 149310.68 | 0.029 |
| Pruned model (a =.05) |  | 149926.99 | 149287.81 | 0.028 |
| Step-up model |  | 149911.67 | 149251.53 | 0.027 |

Note; Panel-GVAR: Panel graphical vector autoregression; BIC: Bayesian information criterion; AIC: Akaike information criterion: RMSEA: root mean square error of approximation. We first generated a base panel-GVAR with all edges, then performed model edges pruning to improve robustness against false positive findings, and then gradually reintroduced edges in a final step-up model with optimal fit.
